# Incidence and predictors of kyphotic deformity following resection of cervical intradural tumors in adults: a population-based cohort study

**DOI:** 10.1007/s00701-020-04416-4

**Published:** 2020-06-16

**Authors:** Charles Tatter, Alexander Fletcher-Sandersjöö, Oscar Persson, Gustav Burström, Per Grane, Erik Edström, Adrian Elmi-Terander

**Affiliations:** 1 Department of Neurosurgery, Karolinska University Hospital, Elite Hotel Carolina, 4th floor, 171 76 Stockholm, Sweden; 2 Department of Clinical Neuroscience, Karolinska Institutet, Stockholm, Sweden; 3 Department of Neuroradiology, Karolinska University Hospital, Stockholm, Sweden

**Keywords:** Cervical spine, Kyphosis, Laminectomy, Laminoplasty, Intradural tumor, Spinal cord tumor

## Abstract

**Background:**

The first line of treatment for most cervical intradural tumors is surgical resection through laminotomy or laminectomy. This may cause a loss of posterior pulling force leading to kyphosis, which is associated with decreased functional outcome. However, the incidence and predictors of kyphosis in these patients are poorly understood.

**Object:**

To assess the incidence of posterior fixation (PF), as well as predictors of radiological kyphosis, following resection of cervical intradural tumors in adults.

**Methods:**

A population-based cohort study was conducted on adult patients who underwent intradural tumor resection via cervical laminectomy with or without laminoplasty between 2005 and 2017. Primary outcome was kyphosis requiring PF. Secondary outcome was radiological kyphotic increase, measured by the change in the C2–C7 Cobb angle between pre- and postoperative magnetic resonance images.

**Results:**

Eighty-four patients were included. Twenty-four percent of the tumors were intramedullary, and the most common diagnosis was meningioma. The mean laminectomy range was 2.4 levels, and laminoplasty was performed in 40% of cases. No prophylactic PF was performed. During a mean follow-up of 4.4 years, two patients (2.4%) required delayed PF. The mean radiological kyphotic increase after surgery was 3.0°, which was significantly associated with laminectomy of C2 and C3. Of these, C3 laminectomy demonstrated independent risk association.

**Conclusions:**

There was a low incidence of delayed PF following cervical intradural tumor resection, supporting the practice of not performing prophylactic PF. Kyphotic increase was associated with C2 and C3 laminectomy, which could help identify at-risk patients were targeted follow-up is indicated.

**Electronic supplementary material:**

The online version of this article (10.1007/s00701-020-04416-4) contains supplementary material, which is available to authorized users.

## Introduction

Spinal intradural tumors are benign or malignant growths that arise in or around the spinal cord. They account for 2–4% of all primary central nervous system tumors [1] and can give rise to symptoms such as sensorimotor disturbances and urorectal dysfunction [2]. The first line of treatment for most cervical intradural tumors is surgical resection [3] where the spinal canal is usually accessed via a posterior approach that includes detachment of paraspinal muscles and removal of the spinous process and lamina as well as associated ligaments including the interspinous ligament and ligamentum flavum [4, 5]. As a consequence, extensor muscle force may be reduced. Experimental models and finite element studies have shown that the majority of the axial load transmission in the cervical spine goes through the posterior columns [6]. Loss of extensor force may therefore lead to a forward shift of the axial load transmission, predisposing the patient to development of cervical kyphosis [7, 8]. Highlighting this, several authors have reported a high incidence of postoperative kyphosis after intradural tumor surgery [9]. This has been shown to impair functional outcome [5, 10, 11] and may also require later stabilization with posterior fixation [12, 13]. Despite this, predictors of kyphotic deformity and incidence of posterior fixation following resection of cervical intradural tumors are poorly defined.

The aim of this study was to assess the incidence and predictors of long-term kyphosis following resection of cervical intradural tumors, evaluating the need for delayed posterior fixation surgery and describing situations where prophylactic stabilization may be indicated.

## Methods

### Patient selection and study setting

All adult patients (≥ 15 years) who underwent cervical laminectomy (where lamina was removed and not reinstated) or laminectomy with laminoplasty (where lamina was removed but reinstated using microplates and screws) and intradural tumor resection between 2005 and 2017 were eligible for inclusion. Exclusion criteria were previous cervical laminectomy and simultaneous laminectomy of the thoracic spine; the latter was to avoid potential confounding effects of laminectomy at the cervicothoracic junction in accordance with previous literature [14]. The study hospital is a publicly funded and owned tertiary care center serving a region of roughly 2 million inhabitants and is the only neurosurgical center in the region. Patients were identified using the surgical management software Orbit (EVRY Healthcare Systems, Solna, Sweden) and were, as part of a previous study, cross-referenced with the national cancer registry to ensure that all eligible patients were included [2]. Medical records and imaging data from digital hospital charts were retrospectively reviewed using the health record software TakeCare (CompuGroup Medical Sweden AB, Farsta, Sweden). The study was approved by the Regional Ethical Review Board (Dnr: 2016/1708-31/4) who waived the need for informed consent.

### Surgical technique and follow-up

The operations were performed by either of three senior consultant neurosurgeons as the primary attending. With the patient in prone position, a posterior midline approach was performed. Laminectomy was then conducted using an ultrasonic bone scalpel (from 2012 to 2017) (Misonix Inc., Farmingdale, NY, USA) or a high-speed diamond bit drill and Kerrison rongeur (between 2005 and 2011). Under the microscope, the dura was incised, and the arachnoid was dissected sharply, allowing exposure of the tumor. For extramedullary tumors, the cranial and caudal poles were identified, and the tumor was dissected from surrounding structures. For intramedullary tumors, a midline myelotomy was performed using a diamond knife, and the tumor was then dissected sharply or removed using an ultrasonic aspirator (Sonopet, Stryker, USA). When visual gross total resection was achieved, the myelotomy was closed and sutured [2]. Watertight dural closure was performed in all cases. When laminoplasty was performed, the laminae were repositioned using microplates (CMF Medicon Surgical Inc., Jacksonville, FL, USA). The soft tissue layers were then sutured individually to close the wound. Following surgery, all patients were subjected to long-term clinical and radiological follow-up in accordance with local protocols, which differed slightly depending on tumor type and histopathological grade. Those who presented with pain or neurological deficit due to kyphosis were considered for posterior fixation surgery.

### Radiological assessment

**Fig. 1:**
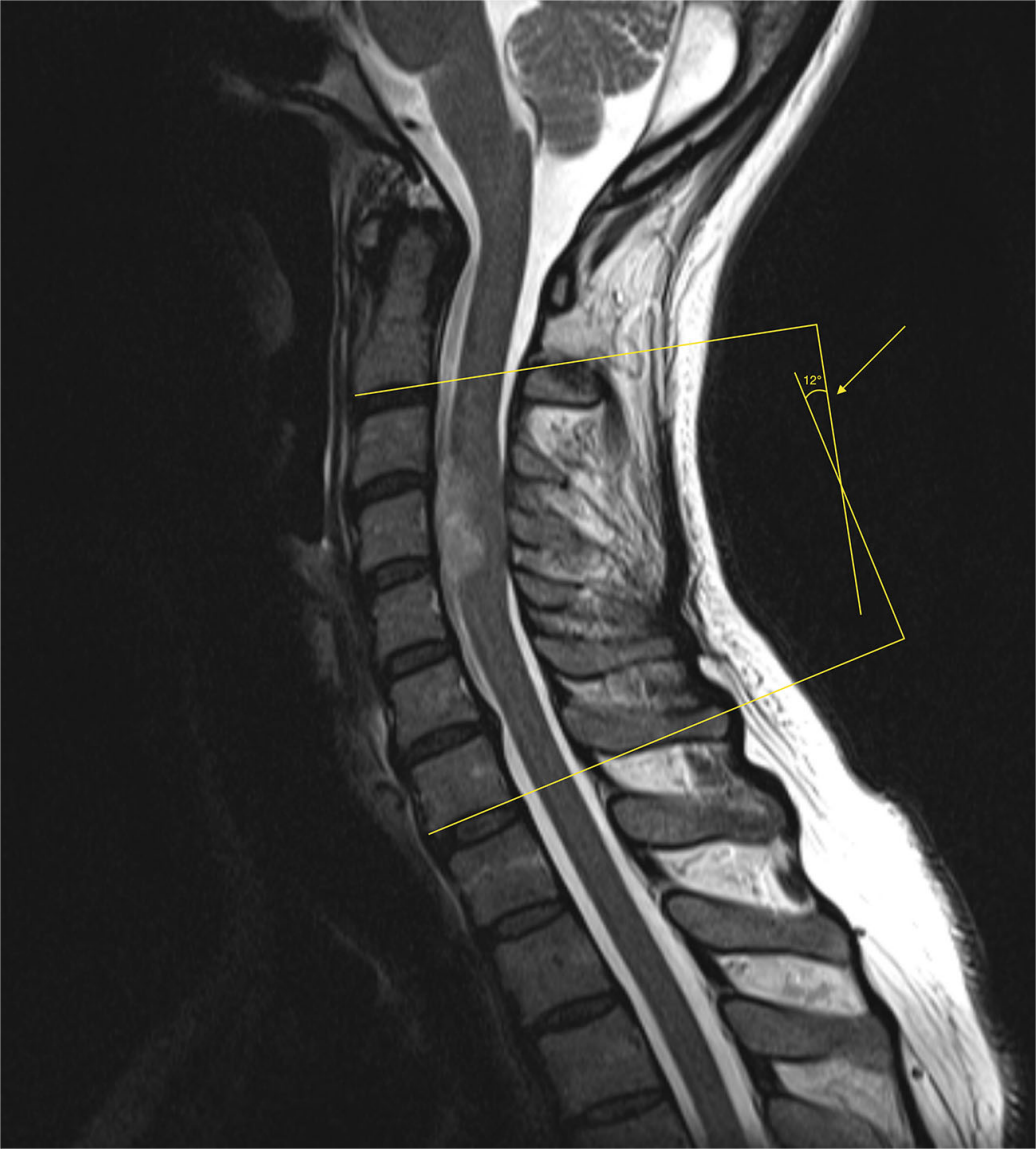
Measurement of a 12° C2–C7 Cobb angle from a sagittal T2-weighted magnetic resonance image

Calculation of cervical lordosis and kyphotic increase was performed on midline sagittal views of supine magnetic resonance images (MRI) using PACS (Picture Archiving and Communication System, IDS7, Sectra AB, Linköping, Sweden). Cervical lordosis was measured with the C2–C7 Cobb angle [11], which measures the angle between the lower endplates of C2 and C7 (Fig. 1). Kyphotic increase was defined as the difference in C2–C7 Cobb angle between the preoperative and long-term follow-up MRI (“delta-cobb”). For patients that required a delayed stabilization procedure, the last MRI prior to surgery was used.

### Statistics

For descriptive purposes, continuous data are presented as mean (standard deviation), and categorical data, as numbers (proportion). Shapiro-Wilk test was used to test for normality of distribution. Two statistical models were used. First, McNemar’s test (dichotomized data) and Wilcoxon signed-rank test (continuous data) were used to test for consistent differences between pre- and postoperative functional status. Secondly, a univariate linear regression was conducted to assess predictors of radiological kyphotic increase. For this, delta-cobb was used as the continuous dependent variable where an increase correlated to a larger degree of kyphotic increase. Factors that showed a trend toward significance in the univariate analysis (*p* < 0.1) were then entered into a step-down multivariable linear regression analysis to determine independent risk factors. In the step-down model, the least significant variable was sequentially eliminated until only significant variables remained. Listwise deletion was used to handle missing data. The statistical significance level was set to *p* < 0.05. All statistical analyses were conducted using SPSS (IBM Corp. IBM SPSS Statistics, Version 25.0, 2017. Armonk, NY: IBM Corp.).

## Results

### Participants and descriptive data

A total of 112 patients met the inclusion criteria. Among these, 5 patients were excluded due to previous cervical laminectomy, 13 were excluded due to simultaneous laminectomy of the thoracic spine, and 10 were lost to follow-up. Thus, 84 patients were included in the study and constitute the study cohort. Thirty-seven (44%) of the patients were male, and the mean age was 52 years. The mean preoperative C2–C7 Cobb angle was 14 ± 15° lordosis. The mean preoperative modified McCormick scale was 1.8 ± 0.7, and the most common symptoms were pain (*n* = 50, 60%) and sensory deficit (*n* = 44, 52%). Six patients (7%) had neurofibromatosis (Table 1).

**Table 1:** Baseline characteristics and treatment outcomes

Variable	Value (*n* = 84)
Baseline data	
Male sex	37 (44%)
Age (years)	52 ± 15
Prior cervical radiation	1 (1.2%)
Neurofibromatosis	6 (7%)
Body mass index (BMI)	26 ± 4.6
C2–C7 angle	14 ± 15° lordosis
Symptoms	
Modified McCormick scale	1.8 ± 0.7
Motor deficit	38 (45%)
Sensory deficit	44 (52%)
Pain	50 (60%)
Treatment data	
Intramedullary tumor	20 (24%)
Dumbbell tumor	26 (31%)
Tumor extent (levels)	2.1 ± 1.0
Method	
Laminectomy	50 (60%)
Laminoplasty	34 (40%)
Laminectomy extent (levels)	2.4 ± 1.0
Including C1	23 (27%)
Including C2	25 (30%)
Including C3	36 (43%)
Including C4	36 (43%)
Including C5	34 (40%)
Including C6	35 (42%)
Including C7	18 (21%)
Joint resection	5 (6.0%)
Prophylactic posterior fixation	0 (0%)
Adjuvant radiation	1 (1.2%)
Outcome data	
Follow-up time (years)	4.4 ± 3.3
Modified McCormick scale	1.6 ± 0.7
Cervical tumor growth or recurrence	8 (10%)
C2–C7 angle	11 ± 18° lordosis
Delta-cobb	3.0 ± 12° kyphotic increase
Cervical reoperation	5 (6.0%)
Renewed tumor resection	2 (2.4%)
Wound revision (infection)	1 (1.2%)
Posterior fixation	2 (2.4%)
Time to posterior fixation (years)	1.0 and 1.2

Data presented as mean (standard deviation) or number (proportion)

### Treatment

The mean tumor extent was 2.1 ± 1.0 levels, and 26 (31%) were dumbbell shaped. Twenty (24%) of the tumors were intramedullary. The mean laminectomy range was 2.4 ± 1.0 levels. Laminoplasty was performed in 34 cases (40%), and facet joint resection, in 5 (6.0%). Prophylactic posterior fixation was not performed in any patient (Table 1). The most common histological diagnosis was meningioma (*n* = 26, 31%), followed by schwannoma (*n* = 25, 30%) and ependymoma (*n* = 11, 13%) (Table 2).

**Table 2:** Pathology report

Diagnosis	Value (*n* = 84)
Ependymoma	11
Hemangioblastoma	4
Meningioma	26
Neurofibroma	9
Schwannoma	25
Other	11
Intradural chordoma	1
Hemangiopericytoma	1
Histology inconclusive	2
Intramedullary lipoma	2
Neurilemmoma	1
Neurothekeoma	1
Dermoid	1

### Outcome data: clinical parameters

The mean follow-up time was 4.4 ± 3.3 years. During this time, two patients (2.4%) underwent delayed posterior fixation due to kyphosis. Tumor growth or recurrence occurred in eight (10%) patients, of whom two underwent renewed tumor resection (both schwannomas) and one received adjuvant radiotherapy (a suspected malignant intramedullary tumor with inconclusive histology) (Table 1).

Compared to the preoperative status, surgery was associated with a significant decrease in modified McCormick scale (*p* = 0.003), motor deficit (*p* < 0.001), and pain (*p* < 0.001) (Supplementary table 1). Three patients who did not undergo posterior fixation developed neck pain in the absence of significant kyphotic increase and were managed conservatively.

### Outcome data: kyphosis

**Fig. 2:**
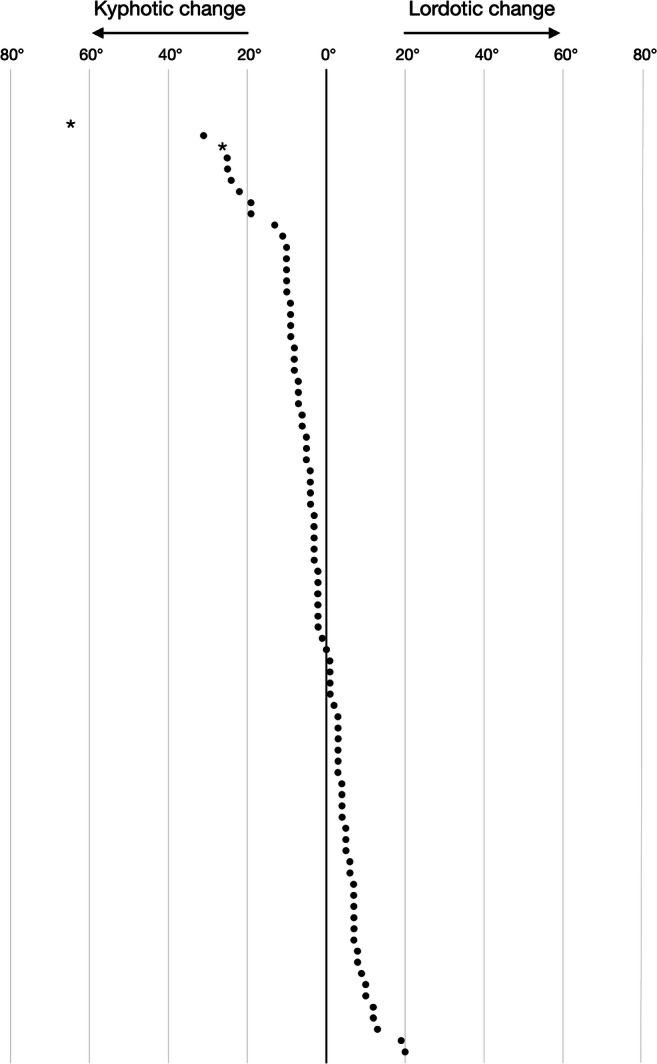
Plot point graph, with each dot representing an individual patient, showing delta-cobb following laminectomy and intradural tumor resection for our cohort. The stars (full width asterisks) mark the two patients who underwent delayed posterior fixation due to symptomatic kyphosis

**Fig. 3:**
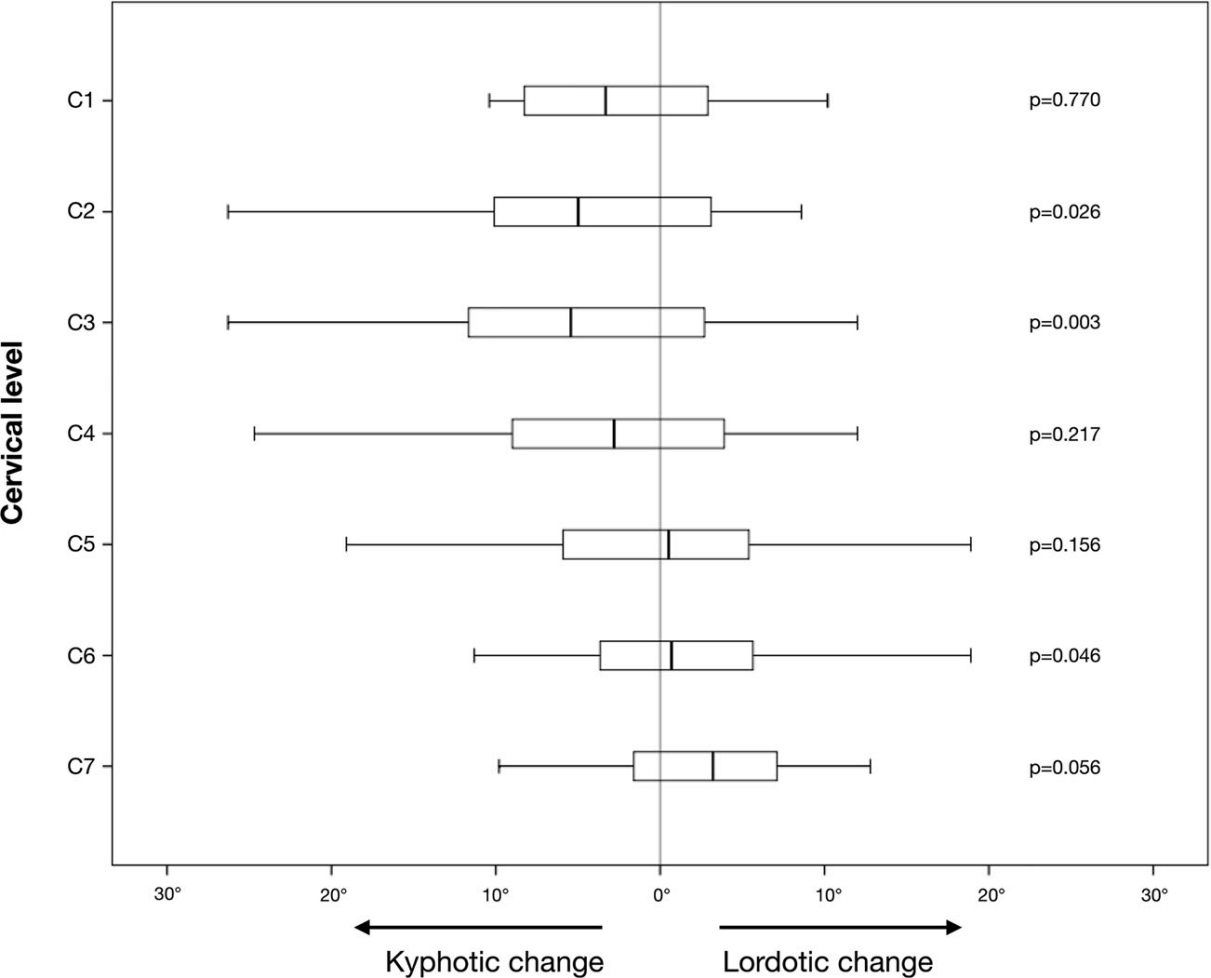
Box plots showing delta-cobb, following laminectomy and intradural tumor resection, depending on the level of laminectomy. *p* values are from a univariate linear regression model using delta-cobb as the dependent variable and each level of laminectomy as the explanatory variable

The mean kyphotic increase (delta-cobb) was 3.0 ± 12° (Table 1). Some degree of kyphotic increase (delta-cobb > 0°) was seen in 47 (56%) patients (Fig. 2). For these patients, the mean kyphotic increase was 10 ± 11°. For the eight patients with tumor growth or recurrence, the mean kyphotic increase was 2.5 ± 9.7°. Laminectomy at higher cervical levels (C1–C4) was more frequently associated with a kyphotic increase while laminectomy of lower levels (C5–C7) was more often associated with a lordotic change (Fig. 3).

In the univariate linear regression model predicting kyphotic increase, laminectomy of C2 (*p* = 0.026, *R*^2^ = 0.049) and laminectomy of C3 (*p* = 0.003, *R*^2^ = 0.100) were identified as significant risk factors (Table 3). In the multivariable model, laminectomy of C3 demonstrated independent risk association (*p* = 0.004). Laminectomy, as opposed to laminoplasty, was not significantly associated with an increased risk for postoperative kyphosis (Table 4).

**Table 3:** Predictors of increased kyphosis: univariate regression analysis

Variable	Univariate *p* value
Age	0.067
Male sex	0.962
Preoperative modified McCormick scale	0.345
Intramedullary tumor	0.544
Dumbbell tumor	0.861
Preoperative C2–C7 angle	0.377
Body mass index (BMI)	0.080
Gross total resection	0.682
Tumor extent	0.791
Laminectomy range (count)	0.445
Laminectomy including C1	0.770
Laminectomy including C2	*0.026*
Laminectomy including C3	*0.003*
Laminectomy including C4	0.217
Laminectomy (i.e., not laminoplasty)	0.618
Joint resection	0.761

Italic text in the *p* value column indicates a statistically significant correlation (*p* < 0.05)

**Table 4:** Independent predictors of postoperative kyphosis. Final results from the step-down multivariable logistic regression analysis

	Univariate *p* value	*R*^2^	Multivariable *p* value
Included variable			
Laminectomy including C3	*0.003*	0.100	*0.004*
Excluded variables			
Laminectomy including C2	*0.026*	0.059	0.084
Body mass index (BMI)	0.080	0.037	0.090
Age	0.067	0.040	0.195

Italic text indicates a statistically significant correlation (*p* < 0.05)

### Subgroup analysis: posterior fixation

**Fig. 4:**
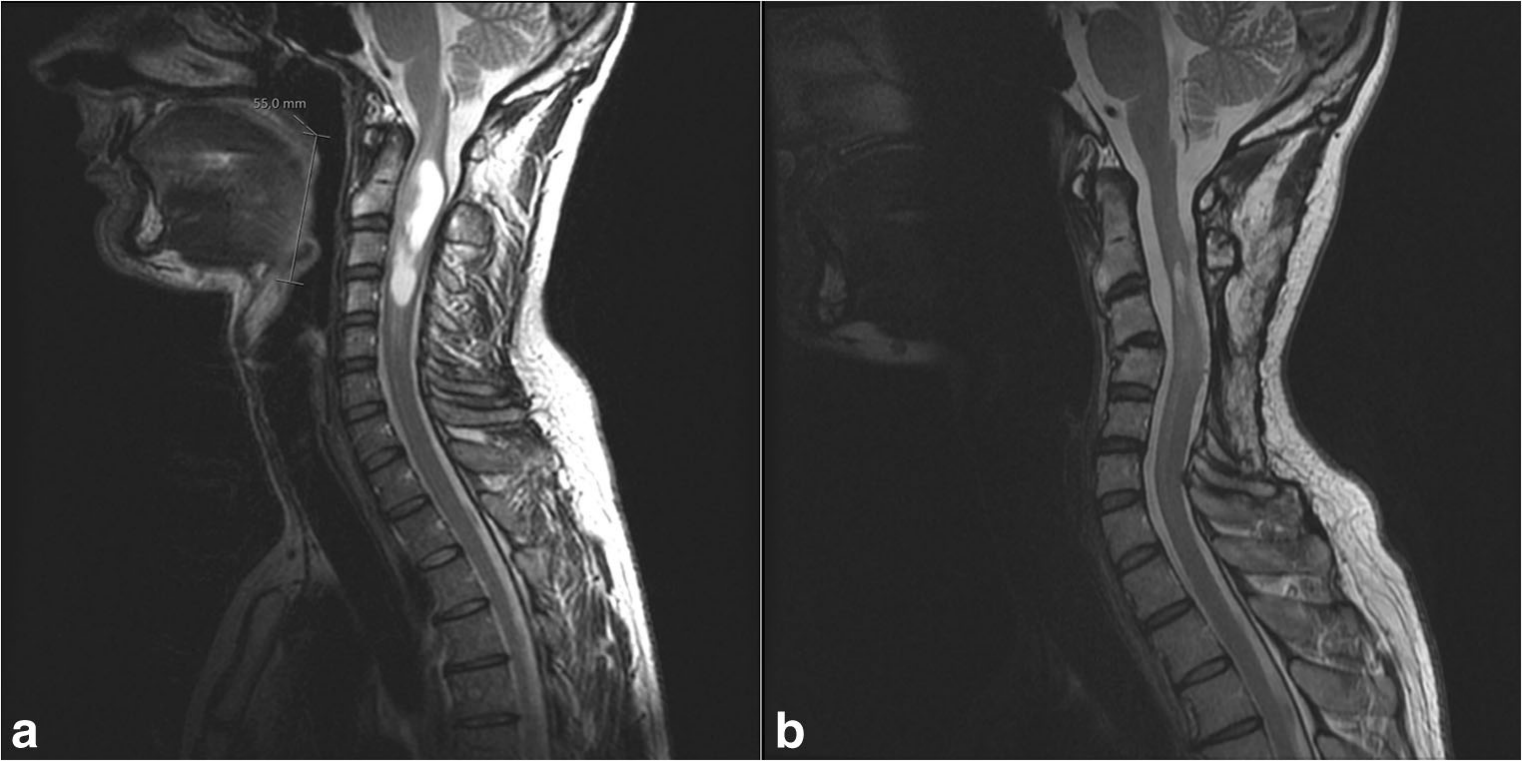
Pre- (**a**) and postoperative (**b**) magnetic resonance image showing kyphosis following cervical laminectomy

**Fig. 5:**
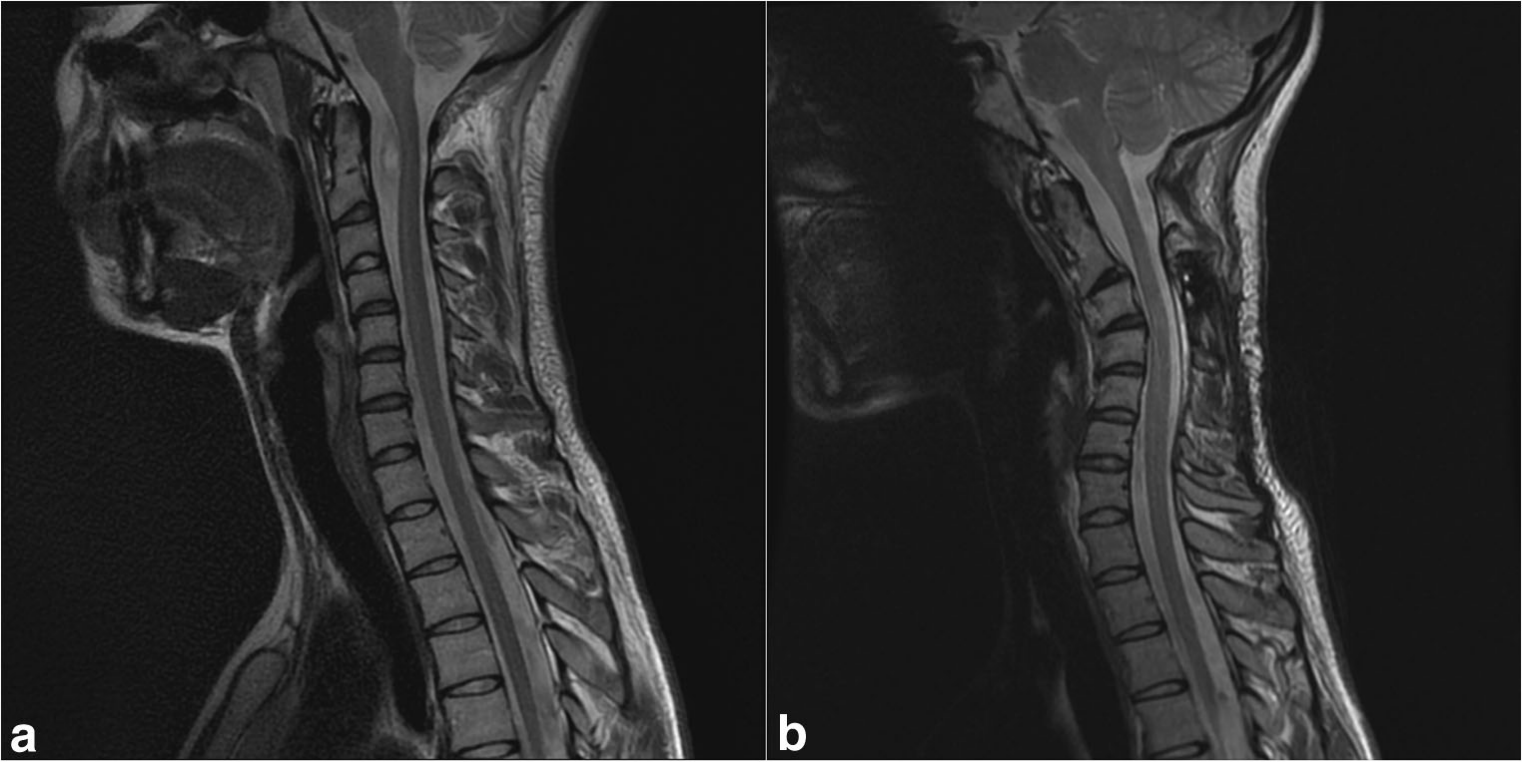
Pre- (**a**) and postoperative (**b**) magnetic resonance image showing kyphosis following cervical laminectomy

Two patients required delayed posterior fixation due to kyphosis. The first patient was a 38-year-old male with an intramedullary ependymoma who underwent tumor resection and laminectomy of C3–C4. He had a preoperative C2–C7 Cobb angle of 28° lordosis. Following surgery, he gradually developed cervicalgia and restrained cervical mobility, and MRI revealed a kyphotic increase (delta-cobb) of 65° (Fig. 4). He underwent posterior fixation of C2–C5 13 months after initial tumor resection, with complete symptom resolution. The second patient was a 21-year-old female with a neurofibroma who underwent tumor resection and laminoplasty of C1–C3. She had a preoperative C2–C7 Cobb angle of 10° kyphosis. Following surgery, she developed cervicalgia, and MRI revealed a kyphotic increase of 26° (Fig. 5). For this, she underwent posterior fixation of C2–C5 15 months after initial tumor resection, with satisfactory outcome.

## Discussion

The aim of this study was to assess the incidence of posterior fixation following resection of cervical intradural tumors, as well as predictors of radiological kyphotic increase. To the best of our knowledge, this is the largest study of its kind and contributes new findings that are important for patient management and future study design.

The mean preoperative C2–C7 Cobb angle was 14° lordosis, which is considered normal according to a recent metanalysis of cervical lordosis in asymptomatic individuals [15]. During a mean follow-up time of 4.4 years, two (2.4%) patients required a delayed posterior fixation. This incidence is lower compared to previous studies of cervical intradural tumors treated with laminectomy and tumor resection. In a study of 34 patients, Katsumi et al. reported that 9% required a posterior fixation within an undefined follow-up time [16]. In another study of 45 adult patients with cervical intradural tumors, of whom 13 underwent prophylactic fusion, 5 (16 %) of the remaining patients also required a delayed posterior fixation within a minimum follow-up time of 24 months [14]. It should be noted that both studies reported a larger laminectomy range than ours, with Sciubba et al. reporting a mean of 2.6 levels and Katsumi et al. reporting a mean of 3.2 levels (as compared to our mean of 2.4 levels). Moreover, we performed laminoplasty in 40% of cases as compared to Sciubba et al. who performed laminoplasty in 19% and Katsumi et al. who did not perform laminoplasty at all [16]. Thus, we report a 2.4% incidence of delayed posterior fixation following initial intradural tumor resection, which compares favorably to the existing literature and might be due to shorter laminectomy range and a higher degree of laminoplasty.

We found a mean kyphotic increase (delta-cobb) of 3.0° and that 56% of patients showed some form of kyphotic increase (delta-cobb > 0°). Generally, we found that our cohort showed a normal distribution around zero with a few outliers where there was a large kyphotic increase (Fig. 2). We believe this highlights the fact that most patients do not run a risk of kyphosis but that there is a subset of at-risk patients that warrant early identification. Alluding to this, laminectomy of C2 and C3 was identified as a significant risk factor for kyphotic increase. This is supported by two previous studies that found intradural tumor resection and laminectomy of C2 to be associated with upper-level cervical kyphosis [17] and cervical instability [16]. Further highlighting the clinical significance of these results, the two patients who required a delayed posterior fixation in our cohort had undergone laminectomies including C2 and/or C3. In contrast, laminectomy of lower levels (C5–C7) was more often associated with a lordotic change (Fig. 3). This finding has previously been observed in studies of spinal deformity following surgery for spinal cord tumors [17, 18]. The reason behind this may be explained by the findings in a finite element study in which Saito and colleagues demonstrated that the primary cause of post-laminectomy deformity was removal of the spinous processes and the posterior ligaments, causing the originally uniformly distributed stress to be transferred to the facets at the levels of laminectomy. In the post-laminectomy model, they asserted that the gravitational center of the head determined whether the deformity would develop as a kyphosis or increasing lordosis. In the model of upper cervical laminectomy, tensile stress was observed in the posterior direction causing increased pressure on the anterior part of the vertebral bodies and kyphosis. Conversely, a more lordotic pattern developed as the loading point was shifted in the posterior direction, as was the case for laminectomy at the lower cervical spine [19]. This explains why removal of the C7 lamina may result in lordosis of the cervical spine when measured using the C2–C7 angle.

While both C2 and C3 were identified as predictors of postoperative kyphosis in the univariate analysis, C3 was the only one that showed independent risk association in our multivariable analysis. A possible explanation for this may be a bias where C2 laminectomy is avoided if not centered over the tumor and therefore essential to the surgery. In these cases, the adjacent C3 lamina will most often also be removed resulting in a material with very few cases of C2 laminectomy without C3. In our cohort with 25 C2 laminectomies, only 1 was an isolated C2 laminectomy, and 9 were C1–C2 laminectomies. Thus, the remaining 60% (*n* = 15) of C2 laminectomies were combined with a C3 laminectomy. Furthermore, while the spinous process and muscle attachments of C3 in itself are usually small, surgical access to the C3 lamina often requires detaching at least the inferior parts of the muscle insertions on C2. Several studies have emphasized the importance of the extensor muscle force on sagittal balance and that their detachment from C2 causes instability [16, 20–22]. Thus, in line with the proposed mechanism of relative extensor muscle weakness, this detachment of muscles from C2 may suffice in placing the patient at risk for development of cervical kyphosis [23].

In two previous studies of cervical intradural tumor resection, age at operation, preoperative spinal curvature, ≥ 3-level cervical laminectomy, ≥ 4-level cervical laminectomy, and destruction of facet joints were identified as predictors of cervical instability [16] or instability requiring fusion [14]. These were not identified as risk factors in our study. One reason behind this could be the difference in laminectomy range and use of laminoplasty, as described above. It could also be due to a difference in outcome variable. For example, while we analyzed risk factors of radiological kyphosis as a continuous variable, Sciubba et al. analyzed predictors of kyphotic deformity requiring surgical stabilization [14]. This was not possible in our study due to the low incidence of posterior fixation.

We did not find that intramedullary tumor localization increases the risk for kyphosis. Of note, the mean kyphotic increase (delta-cobb) for intramedullary tumors was 4.4° as compared to 2.6° for the remaining tumors. Moreover, intramedullary tumors underwent a mean laminectomy range of 3.2 levels as compared to 2.2 levels in the remaining cohort. Thus, it is possible that the regression analysis was underpowered to show a statistically significant difference between intra- and extramedullary tumors.

We did not find that laminoplasty decreased the risk for kyphosis. While there may be a theorized decreased risk for iatrogenic deformity, the literature has yet to establish conclusive efficacy of laminoplasty over laminectomy in preventing spinal deformity following intradural tumor resection as well as degenerative spinal disorders [5, 18, 24]. Thus, our result is in accordance with the literature.

### Limitations

There is no golden standard for measuring cervical sagittal malalignment. We, and many others [11], used the C2–C7 Cobb angle as we believe it to be a reliable quantification of overall global cervical alignment that does not overlook distal junctional kyphosis [25]. However, other measurements of cervical sagittal malalignment exist, for example T1 slope, sagittal vertical axis, chin-brow vertical angle, and Cobb angle with the surgical area as end points [11, 15, 26]. Moreover, all measurements were performed on MRI obtained in a supine position, which may not be sensitive enough to visualize non-rigid kyphosis. MRI coil placement may also have influenced results. Furthermore, while some have dichotomized their results by employing cut-off values [5, 26], there is no definition of kyphotic change based on the C2–C7 Cobb angle. Lastly, radiologic evidence of kyphosis may not necessarily correlate to clinical outcome. For example, the patient in our cohort with the second highest kyphotic increase did not develop symptoms requiring posterior fixation (Fig. 2). Considering this, the most clinically relevant outcome measurement for future studies may be symptomatic kyphosis requiring stabilization.

## Conclusion

The need for delayed posterior fixation in patients who underwent cervical intradural tumor resection was low, supporting the practice of not performing prophylactic posterior fixation. We found that kyphotic increase was associated with C2 and C3 laminectomy, which could help identify a subset of at-risk patients were targeted radiological and clinical follow-up is indicated.

## Electronic supplementary material

ESM 1 (DOCX 33 kb)
