# Supplementary material for: Incidence and predictors of kyphotic deformity following resection of cervical intradural tumors in adults: a population-based cohort study
Source: Acta Neurochir (Wien). 2020 Jun 16;162(11):2905–13. doi: 10.1007/s00701-020-04416-4 (PMC7550319; doi:10.1007/s00701-020-04416-4)
Supplement: Supplementary file 1 — (DOCX 33 kb) [file 701_2020_4416_MOESM1_ESM.docx]

**Supplementary table 1. Functional outcome: statistics**

| **Variable** | **Pre-operative (n = 131)** | **Post-operative (n = 131)** | **p-value** |
| --- | --- | --- | --- |
| Modified McCormick Scale | 1.8 ± 0.7 | 1.6 ± 0.7 | **0.003** |
| Motor deficit | 38 (45 %) | 19 (23 %) | **< 0.001** |
| Sensory deficit | 44 (52 %) | 36 (43 %) | 0.115 |
| Pain | 50 (60 %) | 28 (33 %) | **< 0.001** |

Data presented as mean (standard deviation) or number (proportion)
Bold text in the p-value column indicates a statistically significant correlation (p < 0.05)
